# Multifunctional protein APPL2 contributes to survival of human glioma cells

**DOI:** 10.1016/j.molonc.2012.08.003

**Published:** 2012-09-05

**Authors:** Beata Pyrzynska, Magdalena Banach-Orlowska, Marta Teperek-Tkacz, Katarzyna Miekus, Grazyna Drabik, Marcin Majka, Marta Miaczynska

**Affiliations:** ^1^ International Institute of Molecular and Cell Biology, Laboratory of Cell Biology, 4 Ks. Trojdena Street, 02-109 Warsaw, Poland; ^2^ Dept. of Transplantation, Jagiellonian University, Medical College, 265 Wielicka Street, 30-663 Cracow, Poland

**Keywords:** Tumor cell biology, Apoptosis, Gene expression, Endocytosis, APPL proteins, HRK

## Abstract

Some endocytic proteins have recently been shown to play a role in tumorigenesis. In this study, we demonstrate that APPL2, an adapter protein with known endocytic functions, is upregulated in 40% cases of glioblastoma multiforme, the most common and aggressive cancer of the central nervous system. The silencing of APPL2 expression by small interfering RNAs (siRNAs) in glioma cells markedly reduces cell survival under conditions of low growth factor availability and enhances apoptosis (measured by executor caspase activity). Long‐term depletion of APPL2 by short hairpin RNAs (shRNAs), under regular growth factor availability, suppresses the cell transformation abilities, assessed by inhibited colony formation in soft agar and by reduced xenograft tumor growth in vivo. At the molecular level, the negative effect of APPL2 knockdown on cell survival is not due to the alterations in AKT or GSK3β activities which were reported to be modulated by APPL proteins. Instead, we attribute the reduced cell survival upon APPL2 depletion to the changes in gene expression, in particular to the upregulation of apoptosis‐related genes, such as UNC5B (a proapoptotic dependence receptor) and HRK (harakiri, an activator of apoptosis, which antagonizes anti‐apoptotic function of Bcl2). In support of this notion, the loss of glioma cell survival upon APPL2 knockdown can be rescued either by an excess of netrin‐1, the prosurvival ligand of UNC5B or by simultaneous silencing of HRK. Consistently, APPL2 overexpression reduces expression of HRK and caspase activation in cells treated with apoptosis inducers, resulting in the enhancement of cell viability. This prosurvival activity of APPL2 is independent of its endosomal localization. Cumulatively, our data indicate that a high level of APPL2 protein might enhance glioblastoma growth by maintaining low expression level of genes responsible for cell death induction.

## Highlights

► APPL2 protein levels are elevated in 40% cases of glioblastoma multiforme.
► Overexpression of APPL2 exhibits cytoprotective effects in glioma cells.
► APPL2 depletion reduces survival and transformation abilities of glioma cells.
► Silencing of APPL2 promotes expression of proapoptotic genes *HRK* and *UNC5B*.

**Abbreviations**
GBM: glioblastoma
NuRD: nucleosome remodeling and deacetylase
RNAi: RNA interference
RTK: receptor tyrosine kinase
siRNA: small interfering RNA
shRNA: short hairpin RNA
TCGA: The Cancer Genome Network
qPCR: quantitative polymerase chain reaction

## 1 Introduction

Recent discoveries indicate that some endocytic proteins are involved in multiple steps of cellular signaling, not only by participating in endocytosis of activated receptors, but also by regulating the magnitude of signaling transmitted to the cytoplasm, and further to the nucleus (Sorkin and von Zastrow, 2009). Deregulation of cellular signaling can lead to changes in gene transcription and may cause neoplastic growth and tumorigenesis. Indeed, the involvement of many endocytic proteins in the pathogenesis of cancer has been reported (Mosesson et al., 2008; Pyrzynska et al., 2009). Two homologous proteins APPL1 and APPL2 (adapter proteins containing pleckstrin homology domain, phosphotyrosine binding domain and leucine zipper motif) represent good examples of proteins actively participating in both endocytosis and cellular signaling (Banach‐Orlowska et al., 2009; Miaczynska et al., 2004; Rashid et al., 2009). In addition, some lines of evidence suggest a possible role of these proteins in cancer development and/or progression.

First, APPL proteins associate with several transmembrane receptors and/or participate in their endocytic trafficking. Among them are receptors for epidermal growth factor–EGF (Jones et al., 2006; Lee et al., 2011; Miaczynska et al., 2004; Zoncu et al., 2009), nerve growth factor–NGF (Lin et al., 2006; Varsano et al., 2006), adiponectin (Mao et al., 2006), follicle‐stimulating hormone–FSH (Dias et al., 2010; Nechamen et al., 2004) and glutamate (Husi et al., 2000). Deregulation of endocytosis and defective degradation of such receptors (in particular receptor tyrosine kinases, RTKs) leads to sustained signaling, changes in cell proliferation or transformation (Haglund et al., 2007). Second, in some cell types APPL proteins can enhance the activity of AKT, one of the key kinases involved in the regulation of tumorigenesis. A high level of active AKT increases cell proliferation, resistance to apoptosis and to hypoxic conditions (Manning and Cantley, 2007). APPL proteins interact with AKT1, AKT2, AKT3 (Mao et al., 2006; Mitsuuchi et al., 1999; Nechamen et al., 2007; Saito et al., 2007; Tan et al., 2010; Yang et al., 2003) and with several components of the AKT signaling pathway, like p110 catalytic subunit of phosphatidylinositol 3‐kinase (PI3K) (Mitsuuchi et al., 1999) and two major targets of AKT: GSK3β and TSC2 (Tan et al., 2010). Both APPL proteins appear to be required for hepatocyte growth factor (HGF)‐induced cell survival and migration via activation of AKT (Tan et al., 2010). Third, APPL proteins are able to translocate to the nucleus and interact with the multiprotein complex NuRD (Miaczynska et al., 2004) responsible for modifying chromatin by histone deacetylation and nucleosome remodeling. Overexpression of APPL1 modulates the composition of NuRD complex containing class I histone deacetylase HDAC1 (Banach‐Orlowska et al., 2009). Class I HDACs have recently emerged as targets for anti‐cancer therapy (Weichert, 2009), however HDAC1 was also proposed as a marker for benign tumors and its loss seems to be linked to enhanced tumor malignancy (Lagger et al., 2010). Fourth, through interaction with Reptin APPL proteins stimulate β‐catenin‐dependent transcription (Rashid et al., 2009), which is known to be overactivated in different types of cancer (MacDonald et al., 2009). Finally, APPL1 may also act as a proapoptotic factor by interacting with DCC (deleted in colorectal cancer) and enhancing the DCC‐induced apoptosis in colorectal cancer cells (Liu et al., 2002).

Taking in consideration the facts mentioned above, we investigated here a potential role of APPL proteins in the regulation of tumor growth. We chose glioma as a tumor model because APPL proteins are abundantly expressed in the brain, where they associate with AKT1 (Tan et al., 2010). Additionally, The Cancer Genome Atlas (TCGA) Research Network recently cataloged major genomic abnormalities in human glioblastoma multiforme (GBM) samples (TCGA, 2008; Verhaak et al., 2010). Many of them result in aberrations of signaling molecules and pathways potentially regulated by APPL proteins, like RTKs (mutational activation or amplification of *EGFR*, *PDGFRA* and HGF receptor *MET*) and AKT (inactivating mutations and deletion of *PTEN*, activating mutations of genes encoding PI3K complex *PIK3CA* and *PIK3R1*, amplification of *AKT3*), highlighting their importance for GBM development and further supporting the choice of glioma as our experimental model.

We report here that the level of APPL2 protein is increased in 40% cases of glioblastoma multiforme, when compared to non‐tumor brain tissue. Silencing of APPL2 leads to reduced viability of glioma cell lines, their decreased transformation abilities and impaired growth *in vivo* as xenografts. In line with these findings, gene expression analysis upon APPL2 depletion revealed a marked increase in expression of proapoptotic *HRK* and *UNC5B* genes, which contribute to the reduced cell survival.

## 2 Materials and methods

### 2.1 Tissue, cell lines and transfections

Paraffin‐embedded sections and snap frozen human non‐neoplastic brain tissue (obtained from seizure lobectomy) and glioblastoma samples were kindly provided by the Brain Tumour Tissue Bank (BTTB; London, Canada) and accompanied with pathological reports. Each patient gave his/her written informed consent on the use of the clinical specimens for research. The study on human tumor samples was approved by the Medical Advisory Committee of the BTTB. Human glioma, epithelial (HeLa and A431) and colon cancer (HCT116 and DLD‐1) cell lines were maintained in DMEM (Dulbecco's modified Eagle's medium) supplemented with 5% fetal bovine serum, l‐glutamine (2 mM), penicillin (100 units/ml) and streptomycin (100 μg/ml). siRNA (7.5 nM) was delivered to LN229 cells using HiPerFect™ reagent (Qiagen), whereas U87MG cells were transfected with 10 nM siRNA using SureFect™ reagent (SABiosciences). For delivery of vectors and plasmids LN229 cells were transfected with FuGENE™ reagent (Roche), U87MG cells with Lipofectamine™ LTX (Invitrogen) and T98G cells with Lipofectamine 2000 (Invitrogen). All transfections were performed according to manufacturers' instructions.

### 2.2 siRNA, shRNA, vectors and lentiviral constructs

Four siRNAs against human APPL2 were used, including two HP Genome Wide siRNAs from Qiagen, APPL2si #1 (cat. no: SI02652174) and APPL2si #2 (cat. no: SI02652188), as well as two Silencer Pre‐designed siRNAs from Ambion, APPL2si #3 (ID no: 26320) and APPL2si #4 (ID no: 140524). Three siRNAs against human HRK (ID no: s194952, s194953, s194954) were purchased from Ambion. The following negative control (non‐targeting) siRNAs were used: cat. no: 1022076 (Qiagen), cat. no: 4390843 and 4390846 (Ambion). For long‐term silencing the Block‐it Lentiviral RNAi Expression System (Invitrogen) was used together with three custom target sequences: APPL2‐sh #1, APPL2‐sh #2 and APPL2‐sh #3 as well as control scrambled (scr.) sequences: APPL2‐scr. #1, APPL2‐scr. #2 and APPL2‐scr #3 (Suppl. Table 1). 293FT cell line was used for production of a replication‐incompetent lentivirus that stably expresses the shRNA of interest from the U6 RNAi cassette, followed by transduction of LN229 cells. All procedures were performed according to manufacturer's instructions. The shRNA sequence targeting lamin was used as positive control for lentiviral production and LN229 cell transduction. LN229 cells were further treated with 3 μg/ml blasticidin S (InvivoGen) for a week, to obtain resistant cell pools with constitutive APPL2 silencing.

In overexpression experiments, pcDNA3‐based constructs of untagged and N‐terminally myc‐tagged APPL2 were used. The human *HRK* gene was synthesized (Epoch Biolabs) and cloned in pcDNA3.1 vector (Invitrogen). 200 ng of purified plasmids were transfected per well of 96‐well plate.

The point mutations were introduced into pcDNA3‐myc‐APPL2 using QuikChange XL site‐directed mutagenesis kit (Stratagene) to generate N308D/M310K and K280E/Y283C/G319R mutants which were verified by sequencing.

### 2.3 Immunohistochemical detection

For immunohistochemical staining of paraffin‐embedded human glioblastoma specimens, sectioned at a thickness of 3 μm, the standard protocol according to DAKO's recommendations was used. The DAKO EnVision + System–HRP, combined with DAB + Substrate‐Chromogen, followed by hematoxylin were employed. For pathological examination the sections were stained only with hematoxylin and eosin (H&E).

### 2.4 Western blotting

Protein extracts from brain tissue and glioblastoma were prepared by tissue homogenization in buffer containing 1% Triton X‐100, 1% sodium deoxycholate, 0.1% SDS, 10 mM Tris (pH 8.0), 140 mM NaCl and protease inhibitor cocktail. LN229 and U87MG cells were lysed in RIPA buffer containing 1% Triton X‐100, 0.5% sodium deoxycholate, 0.1% SDS, 50 mM Tris (pH 7.4), 150 mM NaCl, 0.5 mM EDTA and protease inhibitor cocktail. Protein concentration was measured with BCA Protein Assay Kit (Thermo Scientific). Samples of 10–20 μg total protein were subjected to SDS‐PAGE. Resolved proteins were transferred to nitrocellulose membrane (Whatman), probed with specific antibodies, and detected with either enhanced chemiluminescence or Odyssey infrared imaging system (LI‐COR Biosciences).

### 2.5 Antibodies

Polyclonal anti‐APPL2 (Ab 1296) antibody against C‐terminal peptide was previously described (Miaczynska et al., 2004). Anti‐APPL1 (Ab 5002) and anti‐APPL2 (Ab 4567) antibodies against C‐terminal halves of proteins were raised in rabbits (Eurogentech) and previously described (Rashid et al., 2009). The information concerning other primary antibodies used in Western blotting is included in Suppl. Table 2. Secondary horseradish peroxidase‐conjugated antibodies were from Jackson ImmunoResearch, while secondary fluorophore‐conjugated (IRDye 680 and IRDye 800CW) antibodies used in Odyssey system were from LI‐COR Biosciences.

### 2.6 Cell viability, BrdU incorporation and caspase 3/7 activity assays

Cells were seeded in 96‐well plates (5000 cells per well in 100 μl of medium) and transfected the next day. For the viability assay, the Cell Counting Kit‐8 (CCK‐8, Sigma–Aldrich) was used. CCK‐8 solution (10 μl) was added to each well, incubated for 2 h at 37 °C followed by absorbance measurement at 450 nm (OD_450_). Five samples were measured per point and averaged. The inhibitors of PI3K: LY294002 hydrochloride and wortmannin, as well as apoptosis inductors: retinoic acid *p*‐hydroxyanilide (fenretinide, N‐(4‐hydroxyphenyl)retinamide, HPR), ceramide C6 (N‐hexanoyl‐D‐sphingosine) and inactive control reagent dihydroceramide C6 were purchased from Sigma–Aldrich.

The BrdU incorporation rate was estimated with the Cell Proliferation ELISA, BrdU (Roche). The cells were incubated with BrdU for 5 h before fixing and staining with anti‐BrdU‐POD followed by incubation with substrate solution and luminescence measurement.

For the caspase activity estimation, the Caspase‐Glo 3/7 Assay (Promega) was used. 100 μl of the Caspase‐Glo reagent was added to each well, incubated for 1 h followed by luminescence measurement. Five samples were measured per point and averaged. The values corresponding to the caspase 3/7 activity were normalized according to the cell number. Doxorubicin (Sigma–Aldrich) was used as a positive control for caspase 3/7 activation. Human netrin‐1 was purchased from Axxora and used at concentration 150 ng/ml while human EGF was purchased from PeproTech EC Ltd. and used at concentration 10 ng/ml.

### 2.7 Soft agar assay

The 6‐well plates were first covered with bottom layer of 1% low‐melting point agarose (Sigma–Aldrich), prepared in cell growth medium. The cells (10,000 cells per well) were mixed with 0.33% agarose and plated onto the bottom layer. The agar was solidified for 10 min at 4 °C and covered with 1 ml of medium containing 10% serum. Cells were cultured at 37 °C for 3–4 weeks and supplemented once a week with the fresh medium.

### 2.8 Animal studies

Five NOD‐SCID mice per group were injected subcutaneously with 5 × 10^6^ glioma cells. The tumor growth was monitored by measurement in 3–4 days intervals. After 1 month the animals were sacrificed and the tumors were isolated and weighed. The animal studies were approved by the local ethical committee of the Medical College in Cracow.

### 2.9 Flow cytometry

Distribution of cells in the G1, S, and G2/M cell cycle phases was measured based on propidium iodide (PI) staining using standard flow cytometry method. The cells were fixed in 70% cold ethanol for 1 h, washed and incubated for 30 min in 0.1% sodium citrate in PBS containing RNase (10 μg/ml) and 50 μg/ml PI. Measurements were performed using FACSCalibur instrument (Becton Dickinson). Exactly 10,000 events from each sample were collected in a single cell gate. Aggregates and debris were excluded from the analyses by the creation of the gate on the FL2‐W (transit time) *versus* FL2‐A (total cell fluorescence) cytogram according to the standard procedure (http://www.ucl.ac.uk/wibr/services/docs/cellcyc.pdf). The percentages of cells in the G1, S and G2/M phases were determined based on the frequency distribution of DNA content with CellQuest Pro software (Becton Dickinson).

### 2.10 Quantitative PCR

Cells were seeded in 6‐well plates (300,000 cells per well) and transfected the next day. Three days upon transfection total RNA was isolated with High Pure Isolation Kit (Roche). For cDNA synthesis random nonamers, oligo(dT)_23_ and M‐MLV reverse transcriptase (Sigma–Aldrich) were used according to manufacturer's instructions. For estimation of gene expression two types of StellARrays (Lonza) were used: Human Apoptosis (cat. no: 00188295) and Human Cell Cycle Tox and Cancer (cat. no: 00188299), each containing primer pairs for detection of 94 genes of interest plus 2 controls. Quantitative PCR was performed according to manufacturer's recommendations, using a 7900HT Fast Real‐Time PCR thermocycler (Applied Biosystems). The GPR Data Analysis Tool (Lonza) was used to estimate fold change of gene expression upon APPL2 silencing *versus* control. For validation of gene expression changes the TaqMan^R^ Assays (cat. no: 4448892, Applied Biosystems) were used. The PCR reaction was performed with the TaqMan Gene Expression Master Mix (Applied Biosystems).

For estimation of the expression of UNC5 and netrin family members in LN229 and U87MG cells the specific primers were designed using Primer Express 3.0 software (Applied Biosystems; Suppl. Table 3). The PCR reaction was performed with the Kapa Sybr Fast ABI Prism qPCR Kit (KapaBiosystems). For estimation of *DAPK1* and *GAPDH* expression the TaqMan^R^ Assays were used (as described above). All final products were analyzed on 1.5% agarose gels. As a negative control the reverse transcriptase was omitted during the standard cDNA synthesis protocol. The expression values were obtained at least in duplicate from 3 biological replicates. Relative quantification (RQ) method and Data Assist software (Applied Biosystems) were used to estimate fold change of gene expression upon APPL2 silencing (or overexpression) *versus* control. The data were normalized according to the level of housekeeping genes *ACTB* and *GAPDH*. Only samples with efficient silencing (at least 70% reduction in APPL2 mRNA level) were used in these experiments.

### 2.11 Immunofluorescence

HeLa cells grown on coverslips were fixed with 3% paraformaldehyde for 15 min, permeabilized and blocked in solution I (0.1% saponine, 0.2% gelatin, 0.5% BSA in PBS) for 10 min at room temperature. Washed coverslips were incubated with primary antibodies diluted in solution II (0.01% saponine, 0.2% gelatin in PBS) for 1.5 h followed by incubation with Alexa Fluor‐tagged secondary antibodies for 1 h. The coverslips were washed and mounted onto glass slides using Mowiol (Sigma). Images were acquired with laser‐scanning confocal microscope (Leica TCS SP2 AOBS) using a × 63/1.4 numerical aperture oil immersion objective.

### 2.12 Statistical analysis

The standard deviation is presented on the graphs as error bars. The statistical significance was assessed by the Mann – Whitney test. The *p*‐values were marked with the asterisks on the charts (*p < 0.05, **p < 0.01, ***p < 0.001).

## 3 Results

### 3.1 Upregulation of APPL2 protein levels in human GBM samples

**Figure 1:**
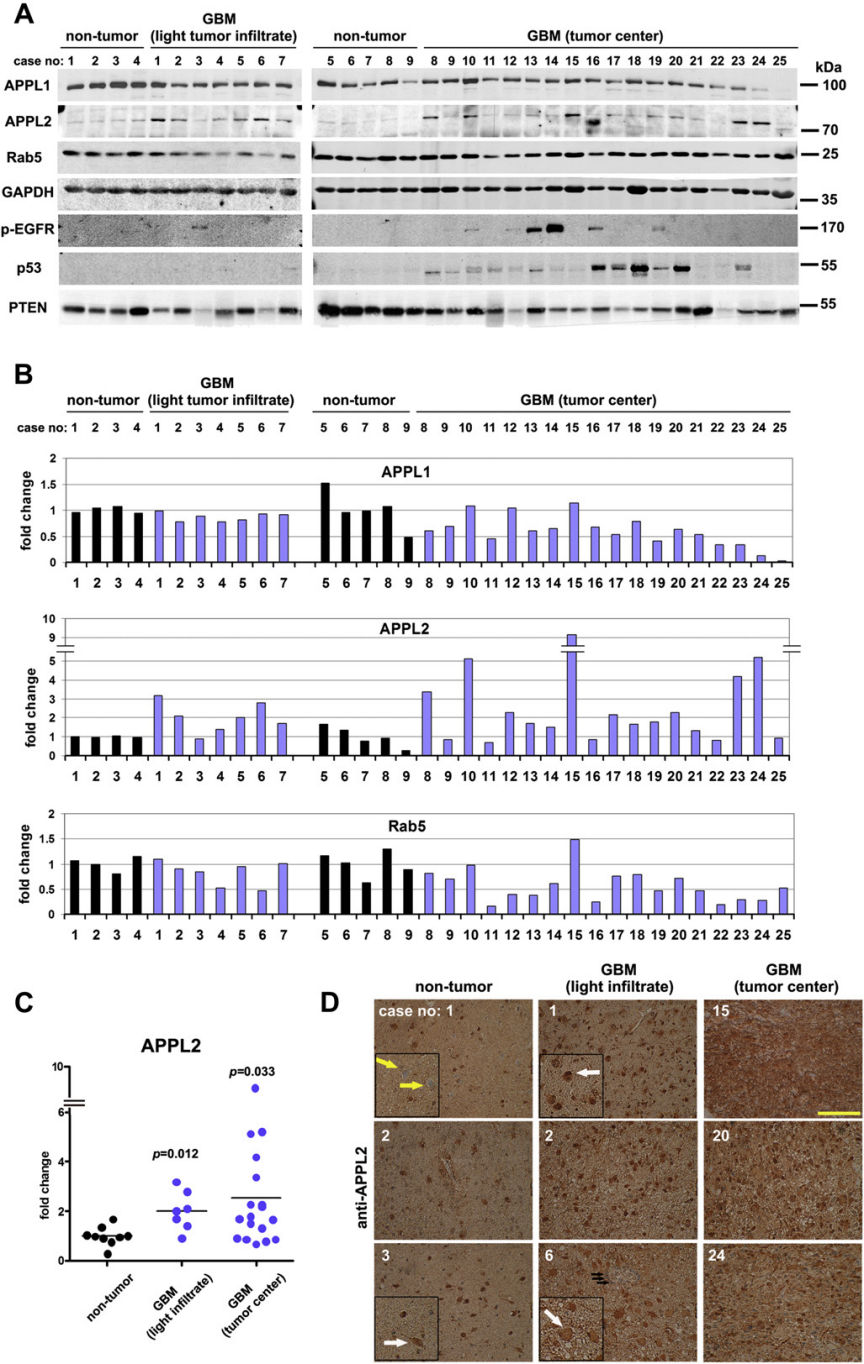
APPL2 protein is upregulated in human GBM samples. (A). Western blot analysis of APPL1, APPL2 (Ab 1296), Rab5, phospho‐EGFR, p53 as well as PTEN, in extracts from non‐neoplastic (referred to as “non‐tumor”) brain tissue and GBM samples. Level of GAPDH was used as a loading control. (B). Quantification of APPL1, APPL2 and Rab5 levels in the samples shown in (A), using infrared imaging. For each sample, the values corresponding to the intensity of bands were first normalized to the values corresponding to bands of GAPDH. Next, the values corresponding to non‐tumor samples were averaged and set as 1. Finally, the fold change for each sample was calculated versus the averaged value mentioned above. (C). Statistical analysis of APPL2 protein level (quantified in panel B) in non‐tumor and GBM samples (light tumor infiltrate and tumor center) presented as a scatter plot. The mean value for non‐tumor tissue group was set as 1. The *p*‐values were estimated using Mann–Whitney test upon comparison of each group of GBM samples versus the group of non‐tumor samples. (D). Immunohistochemical detection of APPL2 protein (Ab 4567) combined with H&E staining in selected samples of non‐tumor tissue as well as GBM cases (case numbers written in an upper left corner on every photo). Insets show higher magnification of images. White arrows mark cells with APPL2 present both in the nucleus and in the cytoplasm; yellow arrows indicate cells lacking anti‐APPL2 staining; black arrows mark endothelial cells. The yellow scale bar = 500 μm.

To test the potential involvement of APPL proteins in growth or progression of brain tumors, we first checked the level of APPL1 and APPL2 in protein extracts isolated from twenty five snap frozen human GBM samples. Two types of samples (each accompanied by a pathological report) were analyzed: i) tissue containing light tumor infiltrate and ii) samples from the tumor center. Additionally, nine samples of non‐neoplastic brain tissue were used as controls. Semi‐quantitative Western blotting analysis using infrared imaging system revealed that APPL2 level was upregulated in twelve GBM samples (over 40% of cases), when compared to non‐neoplastic tissue (referred to as “non‐tumor”, Fig. 1A,B). This upregulation (fold increase between 2 and 9) was present in both types of GBM samples: four samples (out of seven) with light tumor infiltrate (cases no: 1, 2, 5, 6) and eight (out of eighteen) samples of tumor center (cases no: 8, 10, 15, 23, 24 and to a lesser extent cases no: 12, 17, 20). As shown in Fig. 1C, the mean fold change of APPL2 protein level was significantly increased in light tumor infiltrate and in tumor center groups (mean = 2.02 and 2.55, respectively). The higher level of APPL2 protein was noticeable in tumor samples from diverse brain locations and did not correlate with either the recurrent origin of the tumors, the patient's age or sex (Suppl. Table 4), or with the level of proteins known to have altered signaling in GBM, like EGFR or p53 (Fig. 1A). In contrast to APPL2, the levels of homologous APPL1 protein or Rab5 protein did not exceed two‐fold u*p*‐ or downregulation in GBM samples (Fig. 1A, B).

In order to examine the intracellular distribution of APPL2 protein in GBM samples, we performed immunohistochemical (IHC) staining (Fig. 1D). Analysis of the specimens from non‐tumor tissue revealed a population of cells (resembling normal brain cells, white arrow in case no 3) with APPL2 present both in the nucleus and in the cytoplasm, as well as a population of glial cells completely lacking anti‐APPL2 staining (yellow arrows). In the infiltrating tumor cells present in GBM samples (cases no 1 and 2) the cell protrusions and nuclei were strongly stained with anti‐APPL2. Also the infiltrating gemistocytic tumor cells in case no 6 (large cells of irregular shape, white arrow) expressed high levels of APPL2. In contrast, endothelial cells surrounding the blood vessel (black arrows) completely lacked APPL2 staining. Generally, all cases of GBM with upregulated APPL2 levels detected by Western blotting also showed strong IHC anti‐APPL2 staining of tumor cells present both in the tumor center and in the surrounding brain tissue infiltrated by the tumor cells.

We also compared the mRNA expression levels of APPL2 in normal brain and in cancer tissues using the publicly available Oncomine database (www.oncomine.org). Global gene expression patterns in brain tumors of different WHO grades were derived from two studies (Bredel et al., 2005; Sun et al., 2006). According to them, the level of APPL2 mRNA was significantly upregulated in glial brain tumors of various histogenesis and grade, including GBM, oligodendroglioma (OD), anaplastic astrocytoma (AA) and oligoastrocytoma (OA) (Suppl. Fig. 1). No significant alterations in APPL1 mRNA level were detected (not shown).

### 3.2 Silencing of APPL2 expression in glioma cells decreases their viability, anchorage‐independent growth in soft agar and tumor growth *in vivo*

**Figure 2:**
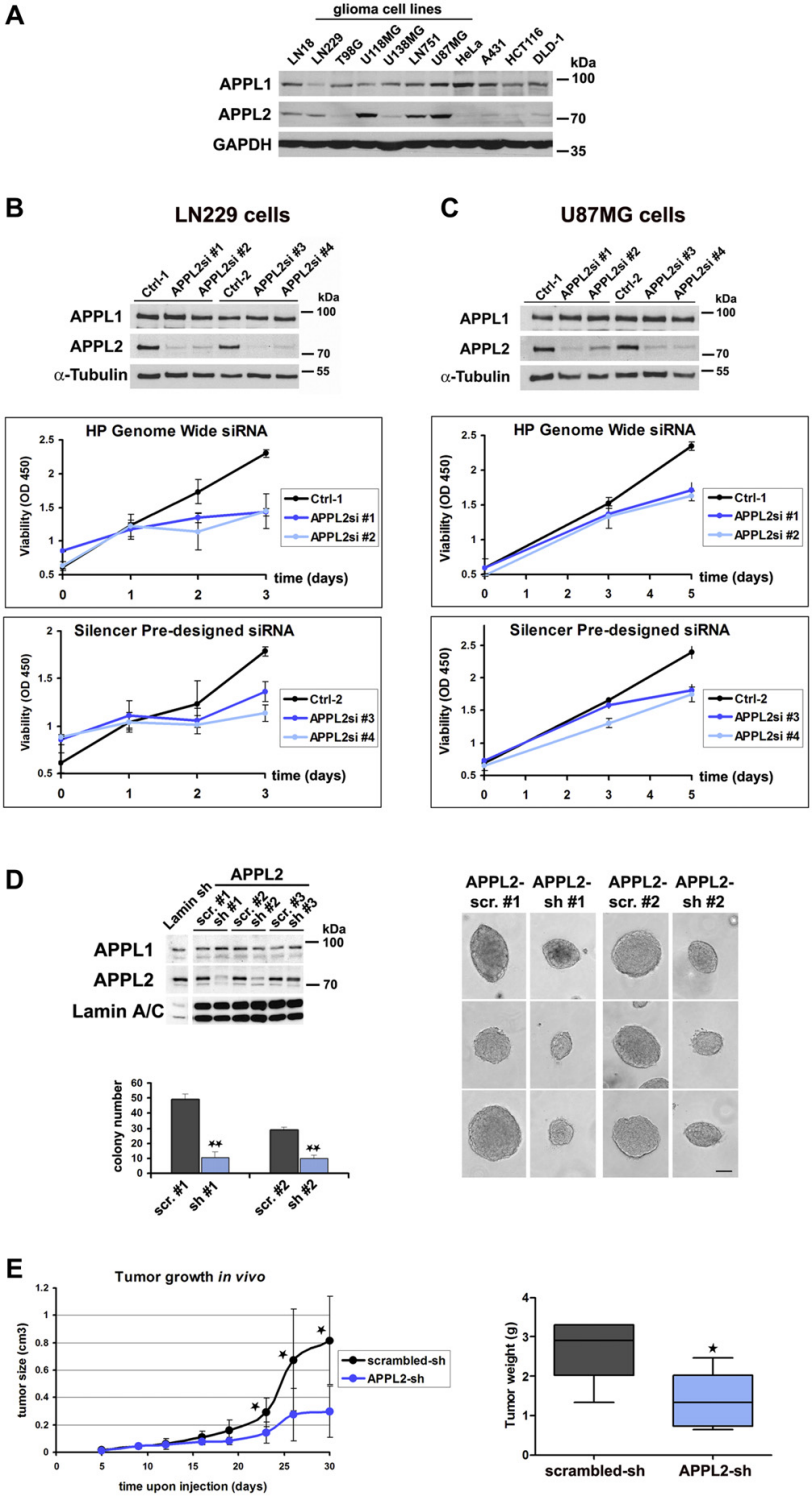
Silencing of APPL2 expression decreases cell viability, growth in soft agar and tumor growth *in vivo*. (A). Western blot analysis of APPL1, APPL2 (Ab 1296) and GAPDH in glioma, epithelial and colon cancer cell lines. (B, C). Specificity and efficiency of APPL2 silencing 3 days upon transfection of glioma cell lines: LN229 and U87MG with different siRNAs targeting APPL2, shown by Western blot (upper panels). Growth curves of glioma cells (lower panels) upon transient silencing of APPL2 expression. Two sources of siRNA (marked on the top of each chart) were used: HP Genome Wide siRNA and Silencer pre‐designed siRNA. (D). Long‐term silencing of APPL2 expression in LN229 cells using the lentiviral system. Three shRNA sequences targeting APPL2 as well as non‐targeting scramble (APPL‐scr.) sequences were used. The Western blot analysis of APPL1, APPL2 and lamin A/C is shown on the left upper panel. The cell pools with the best silencing of APPL2 expression (APPL2‐sh #1 and 2) as well as control cell pools (APPL2‐scr. #1 and 2) were further tested in soft agar assay. The quantification of cell colonies over 500 µm in diameter, formed after 4 weeks of growth in soft agar is shown on the graph (left lower panel). The phase‐contrast photos of the biggest colonies are shown on the right (3 representative photos per each cell pool). The scale bar = 200 µm (E). Pools of LN229 cells (APPL2‐sh #2 and scrambled‐sh #2) were injected subcutaneously into 5 mice and the growth of xenografts was measured during 1 month. The tumor growth curve and final tumor weight after 1 month are shown on the left and right charts, respectively. The *p*‐values were calculated upon comparison of APPL2‐sh with scrambled‐sh and were marked with the asterisk (*p < 0.05).

Upregulation of APPL2 protein levels in over 40% of human GBM samples could either actively contribute to tumor growth or progression, or be a passive consequence of the transformation process. To discriminate between these two possibilities we first measured the effect of APPL2 silencing on the viability of tumor cells. We selected two glioma cell lines, LN229 and U87MG with medium and high endogenous APPL2 protein levels, respectively (comparing to the panel of different cancer cell lines; Fig. 2A).

To silence APPL2 expression we took advantage of four siRNAs with the best silencing potential (estimated in preliminary experiments using Western blotting analysis, data not shown). These siRNAs were chosen from two sources: HP Genome Wide siRNA collection from Qiagen and Silencer pre‐designed siRNAs from Ambion (Fig. 2B, C). We found the statistically significant reduction in the proliferation rate upon silencing of APPL2 expression with each siRNA, in both cell lines grown under low serum availability (blue curves *versus* black (control) curves in Fig. 2B, C and Suppl. Table 5). Moreover, in case of U87MG line this effect was also observed in cells grown under regular serum concentration (Suppl. Fig. 2).

To test whether long‐term silencing of APPL2 expression can affect glioma cell growth we performed experiments with a stringent anchorage‐independent growth assay. We took advantage of a lentiviral silencing system and designed three shRNA sequences targeting APPL2 (Suppl. Table 1). Upon lentiviral transduction we selected the pools of LN229 cells and estimated the efficiency of APPL2 silencing by Western blotting (Fig. 2D, left top panel). Two pools with the best silencing (APPL2‐sh #1 and 2) were next tested in the soft agar assay. The constitutive silencing of APPL2 expression indeed affected the growth in soft agar and reduced the size of colonies when compared to scrambled sequences (Fig. 2D, graph and photographs).

The cell pools were also injected subcutaneously into NOD/SCID mice and their growth was monitored during 1 month (Fig. 2E, left chart). The statistically significant reduction in tumor growth *in vivo* was observed for the cell pool with APPL2 silencing (APPL2‐sh) starting from day 23 post injection. The significant difference in the size of tumors formed from APPL2‐sh and control cells was also confirmed by the estimation of tumor weight at day 30 post injection (Fig. 2E, right chart). Cumulatively, these findings argue that depletion of APPL2 reduces the transformation abilities of glioma cells.

### 3.3 Knockdown of APPL2 induces apoptotic cell death

**Figure 3:**
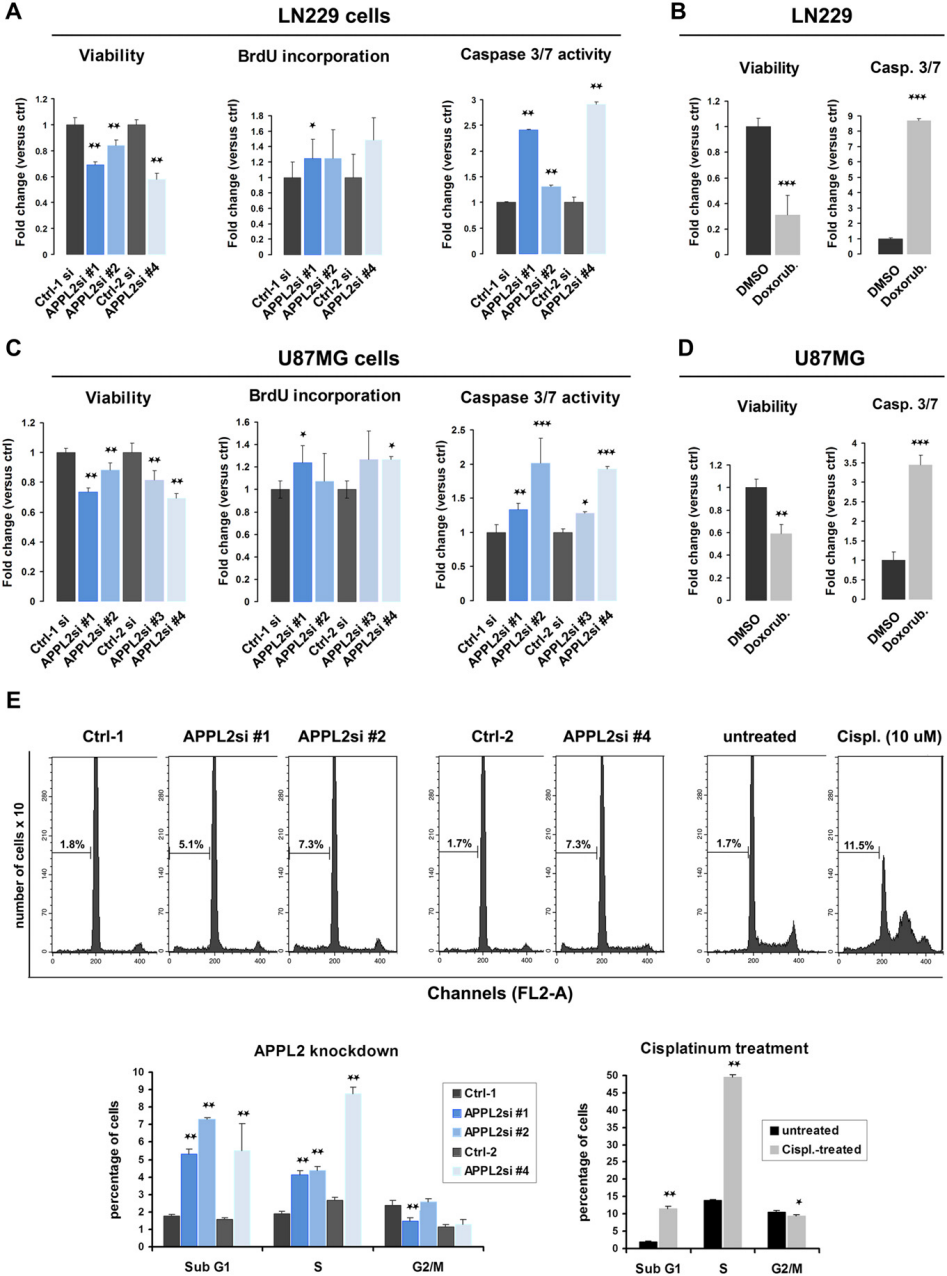
Silencing of APPL2 expression enhances caspase activity. (A). The cell viability (left chart), BrdU incorporation (middle chart) and the activity of caspase 3 and/or 7 (right chart) were estimated in parallel, 4 days upon transfection of LN229 cells with APPL2 siRNAs (the same as in Fig. 2) under conditions of 0.5% serum. The averaged values for each control siRNA were set as 1 and shown as the black bars. (B). The cell viability and the caspase 3/7 activity were estimated like in (A) upon treatment of LN229 cells with 5 µM doxorubicin for 2 days under 5% serum. (C). U87MG cells were transfected with APPL2 siRNAs for 5 days under low serum availability. The cell viability, BrdU incorporation and the caspase 3/7 activity were assayed as in (A). (D). U87MG cells were treated with 10 µM doxorubicin for 2 days followed by viability and caspase 3/7 assays, like in (B). (E). The cell cycle analysis of LN229 cells upon silencing of APPL2 expression for 5 days under low serum availability. Examples of the cell cycle histograms (cells stained with propidium iodide) are shown in the upper panel. The percentage of cells in subG1 phase is marked on the histogram gate. Additionally, two histograms corresponding to untreated LN229 cells in 5% serum and cells treated with 10 µM cisplatinum (cispl.) for 3 days are shown on the right. The averaged percentage of cells in subG1, S and G2/M phases of cell cycle is presented (lower panels). (A–E). The *p*‐values were calculated upon comparison of each APPL2 siRNA to the corresponding control siRNA and were marked with the asterisks (*p < 0.05, **p < 0.01, ***p < 0.001).

To check the proliferation rate and incidence of apoptosis upon APPL2 silencing we measured the BrdU incorporation rate and caspase 3 and/or 7 activity in parallel with cell viability (Fig. 3). In LN229 cells, the decrease in cell viability (Fig. 3A, left chart) corresponded well to a significant increase in caspase activity induced by three APPL2 siRNAs during 4 days of silencing (Fig. 3A, right chart). Although some increase in the proliferation rate, measured by BrdU incorporation, was also detected (middle chart), most of these changes were not statistically significant. As a positive control of apoptosis induction, robust caspase 3/7 activation was induced by the treatment of cells with 5 μM doxorubicin (DNA‐damaging drug; Fig. 3B). In U87MG cells all four siRNAs significantly increased caspase 3/7 activity during 5 days upon APPL2 silencing (Fig. 3C, right chart). Again, doxorubicin was used as control of apoptosis induction (Fig. 3D).

The cell death induction due to the silencing of APPL2 expression was further confirmed in LN229 cells by propidium iodide staining followed by cell cycle analysis by standard flow cytometry. Indeed, upon APPL2 silencing there was a statistically significant increase in the percentage of cells in subG1 phase (corresponding to dead cells) (Fig. 3E, histograms in upper panel). Additionally, an increase in percentage of cells in S phase was also observed (Fig. 3E, left chart in the lower panel), which is in agreement with the results of BrdU incorporation (Fig. 3A, middle chart). As control, the LN229 cells treated with 10 μM cisplatinum (chemotherapy proapoptotic drug) showed increase in both subG1 and S phase (Fig. 3E, right histograms in the upper panel and right chart in the lower panel). In summary, we demonstrated that in both glioma cell lines knockdown of APPL2 decreases cell viability and induces activation of caspases followed by apoptotic cell death.

### 3.4 Cell death induction upon silencing of APPL2 expression is not due to changes in the activity of AKT or GSK3β

**Figure 4:**
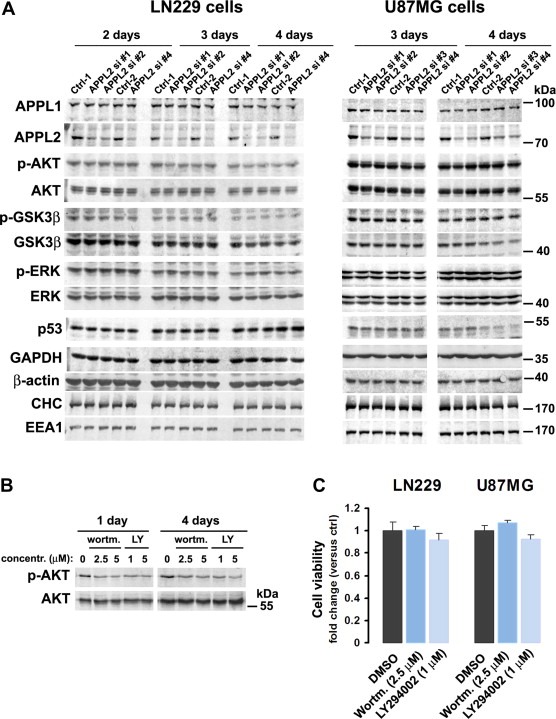
Induction of cell death is not due to inhibition of AKT or GSK3β activity. (A). Western blot analysis of the levels of AKT, GSK3β, ERK and p53 under conditions of low serum availability. Extracts from LN229 cells (left panel) and U87MG cells (right panel) upon silencing of APPL2 expression were analyzed. Levels of GAPDH, β‐actin, clathrin heavy chain (CHC) and EEA1 were used as loading controls. (B). Western blot analysis of the levels of phospho‐AKT (*p*‐AKT) and total AKT upon treatment of LN229 cells with wortmannin (wortm., 2.5 or 5 μM) and LY294002 (LY, 1 or 5 μM) for up to 4 days. (C). Viability of LN229 and U87MG cells was analyzed upon treatment with wortmannin or LY294002 for 4 days under conditions of low serum availability.

To unravel the molecular mechanism responsible for the modulation of glioma cell survival and apoptosis by APPL2, we first examined the pattern of cellular signaling upon knockdown of APPL2 in LN229 and U87MG cells under conditions of low serum availability. AKT and GSK3β activity were previously reported to be modulated by APPL proteins (Schenck et al., 2008) and to affect cell survival or proliferation (Manning and Cantley, 2007). Although we noticed a decrease in the level of active AKT (phospho‐AKT) in LN229 cells at 3 and 4 days upon silencing of APPL2 expression, it was not consistent between different siRNA used (Fig. 4A, left panel and Suppl. Fig. 3). The changes in AKT activity were not visible in U87MG cells, where a very high basal level of phospho‐AKT was detected and remained unaltered upon APPL2 silencing (Fig. 4A, right panel and Suppl. Fig. 3). No changes in phospho‐GSK3β, phospho‐ERK or their total levels were detected in any cell line. Moreover, the level of p53, a known regulator of apoptosis, remained unchanged upon silencing of APPL2 expression.

To verify whether a modest inhibition of AKT could reduce glioma cell survival, we treated LN229 and U87MG cells with 2.5 μM wortmannin or 1 μM LY294002. Even though these treatments were effective (Fig. 4B), no significant changes in cell viability were detected (Fig. 4C). In summary, we conclude that the cell death induction upon silencing of APPL2 expression cannot be due to alterations in the activity of AKT or GSK3β.

### 3.5 Knockdown of APPL2 enhances proapoptotic gene expression

**Figure 5:**
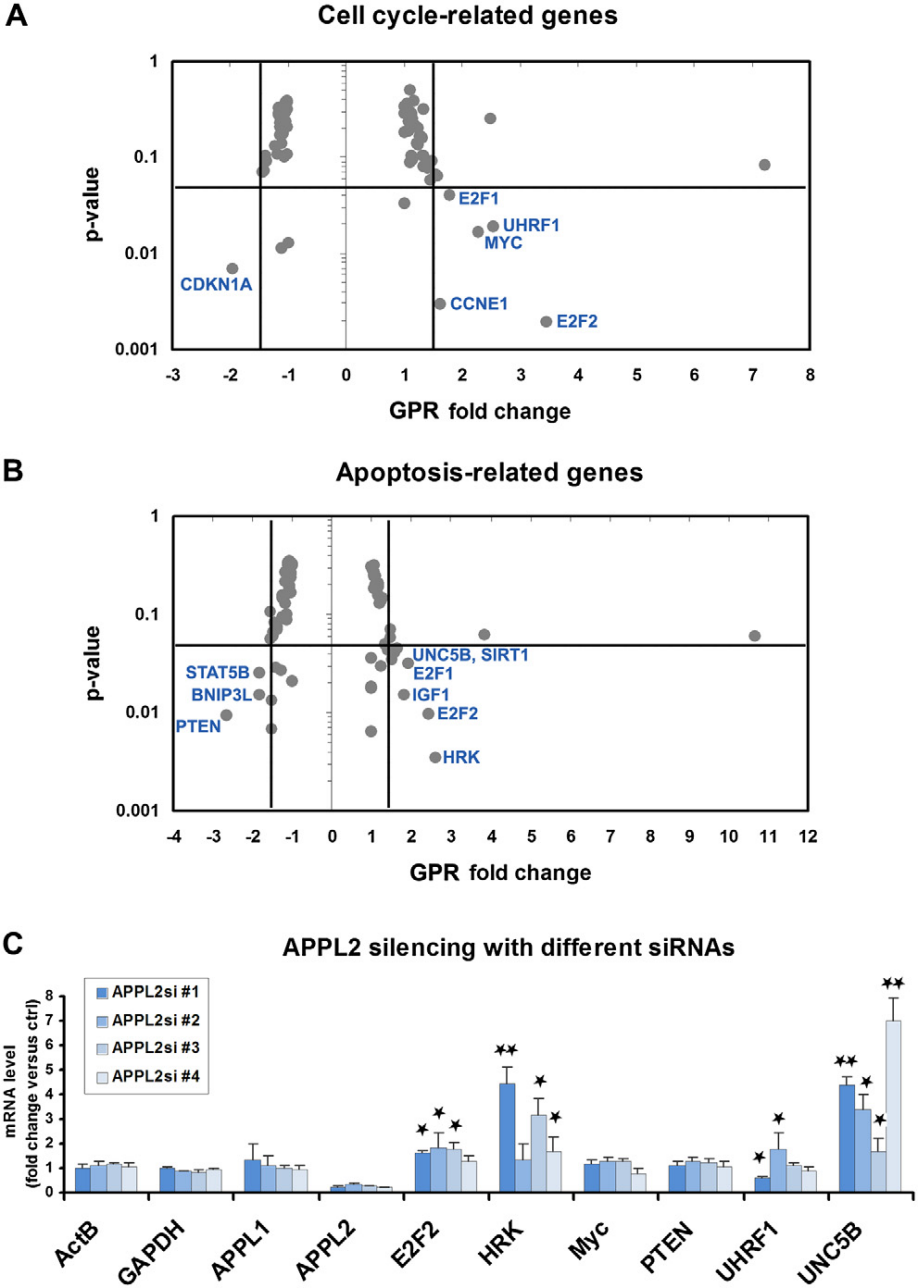
Knockdown of APPL2 level changes expression of cell cycle‐ and apoptosis‐related genes. (A–B). Three days upon knockdown of APPL2 level with APPL2si #1 and 2 (the same siRNAs as in Fig. 2) the gene expression pattern was analyzed using qRT‐PCR in LN229 cells cultured under low serum conditions. Two types of pre‐designed 96‐well arrays were employed: cell cycle‐related (panel A) and apoptosis‐related (panel B). Only genes with fold changes <−1.5 or >1.5 and *p*‐values <0.05 were considered as significantly down‐ or upregulated and their names are mentioned on the charts. (C). Expression of APPL1, APPL2, E2F2, HRK, MYC, PTEN, UHRF1 and UNC5B was validated by qRT‐PCR using the TaqMan gene expression assays in LN229 cells 3 days upon transfection with 4 different siRNAs targeting APPL2 and the corresponding control siRNAs. The *p*‐values were calculated upon comparison of each APPL2 siRNA to the corresponding control siRNA and the significant *p*‐values are marked with the asterisks (*p < 0.05, **p < 0.01).

To further look for the mechanism responsible for modulation of glioma cell survival by APPL2, we examined the pattern of expression of cell cycle‐ and apoptosis‐related genes upon knockdown of APPL2 protein in LN229 cells under conditions of low serum availability, using two siRNAs (APPL2si #1 and 2). Initially we took advantage of the quantitative RT‐PCR StellARray Gene Expression System (Lonza). We found that the mRNA levels of three cell cycle‐related genes (Fig. 5A) and four apoptosis‐related genes (Fig. 5B) as well as two genes involved in both: cell cycle and apoptosis regulation (total nine genes) were significantly elevated (*p*‐value < 0.05, fold change > 1.5). Four significantly downregulated genes were also identified in these experiments (Fig. 5A and B).

We validated these results in an independent set of samples with APPL2 silencing using all four previously mentioned siRNAs. The validation was performed with TaqMan gene expression assays, consisting of a pre‐designed probe and primer pairs for *E2F2*, *HRK*, *MYC, PTEN, UHRF1* and *UNC5B* plus the control housekeeping genes *ACTB* and *GAPDH,* as well as *APPL1* and *APPL2*, to prove good silencing efficiency and specificity of siRNAs targeting APPL2 (Fig. 5C). Among the six selected genes, the most consistent change (obtained with at least three out of four APPL2 siRNAs) was the upregulation of HRK (an activator of apoptosis, which antagonizes anti‐apoptotic function of Bcl2) and UNC5B (a proapoptotic dependence receptor for netrin‐1) mRNA levels (Fig. 5C). Since these proteins are known to regulate cell death, we hypothesized that they may participate in the induction of apoptosis upon APPL2 knockdown.

### 3.6 Induction of *HRK* and *UNC5B* expression contributes to the loss of cell survival upon silencing of APPL2 expression

**Figure 6:**
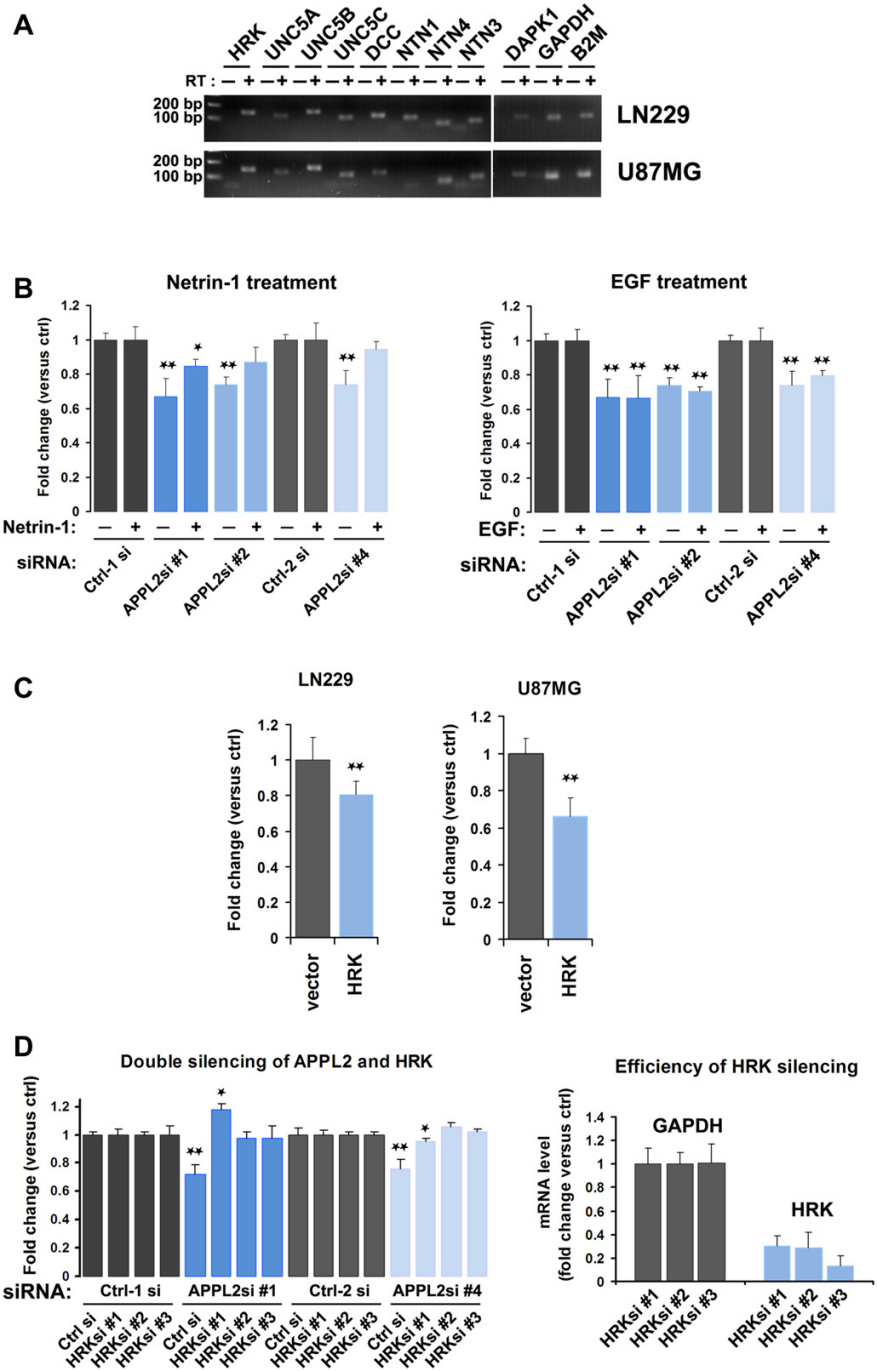
Loss of glioma cell survival depends on the upregulation of HRK and UNC5B expression. (A). The products of RT‐PCR reaction with primers specific for HRK, dependence receptors (UNC5A, UNC5B, UNC5C, DCC), netrin ligands (NTN1, NTN3, NTN4) and DAPK1 were visualized on 1.5% agarose gel. As negative control the reverse transcriptase (RT) was omitted during cDNA synthesis (indicated as “−“). (B). The viability of LN229 cells estimated 3 days upon APPL2 knockdown under limited serum availability (as in Fig. 3). Human netrin‐1 (150 ng/ml) or human EGF (10 ng/ml) was present in the culture medium where indicated by “+” (left and right chart, respectively). (C). The viability of LN229 (left chart) and U87MG cells (right chart) estimated either upon transfection with vector (pcDNA3) or upon overexpression of HRK. (D). The viability of LN229 cells estimated upon double silencing of APPL2 (by APPL2si #1 or #4 at 7.5 nM) and HRK (by three independent siRNAs or non‐targeting siRNA as control, at 7.5 nM each) (left chart). The efficiency of HRK knockdown (right chart) was estimated by quantitative PCR and normalized versus GAPDH expression level. (B–D). The viability of cells transfected with APPL2 siRNAs was normalized to the corresponding control siRNA (set as 1), while the viability of cells overexpressing HRK was normalized to the values corresponding to vector alone. The p‐values were calculated upon comparison of each APPL2 siRNA to the corresponding control siRNA and the significant p‐values are marked with the asterisks (*p < 0.05, **p < 0.01).

To verify the hypothesis about the possible participation of the dependence receptor UNC5B in the loss of cell survival upon knockdown of APPL2 level we first analyzed the expression of different members of UNC5 family (*UNC5A*, *UNC5B*, *UNC5C*), their ligands from the netrin family (*NTN1*, *NTN3*, *NTN4*), another related dependence receptor *DCC* and the signaling molecule of the UNC5B pathway *DAPK1* (Guenebeaud et al., 2010). Almost all genes were expressed in both LN229 and U87MG cells (Fig. 6A) except for *NTN1* in U87MG cells. We therefore concluded that the signaling pathways of the dependence receptors might be functional in both glioma cell lines and UNC5B can be potentially responsible for the loss of their survival upon silencing of APPL2 expression. To verify this hypothesis further we measured the viability of LN229 cells upon APPL2 silencing in the presence of the excess of netrin‐1, the ligand for UNC5B which is known to neutralize its proapoptotic activity (Guenebeaud et al., 2010). Indeed, the presence of netrin‐1 in the culture medium rescued the decrease of LN229 viability (Fig. 6B, left chart) upon APPL2 silencing with three siRNAs shown before to induce caspase 3/7 activity (Fig. 3A). In contrast, EGF, a known activator of the AKT pathway, did not prevent the loss of cell viability upon APPL2 silencing (Fig. 6B, right chart). These data indicate that the regulation of cell survival by APPL2 is at least partially mediated by UNC5B, a conclusion further supported by the fact that overexpression of UNC5B in LN229 (He et al., 2011) and in U87MG cells (Tanikawa et al., 2003) was reported to induce apoptotic cell death.

To study the potential involvement of the proapoptotic protein HRK in the regulation of glioma cell survival we overexpressed HRK in LN229 and U87MG cells and measured their viability 3 days later (Fig. 6C). We observed that HRK has indeed a proapoptotic activity in both cell lines and that the increase in its level is able to induce death of glioma cells. Moreover, simultaneous silencing of APPL2 and HRK expression rescued the loss of cell viability upon transfection with APPL2si #1 and #4 (Fig. 6D, left chart). These data point to the involvement of HRK in death of glioma cells upon knockdown of APPL2.

### 3.7 Endosomal localization of APPL2 is not required for its prosurvival activity and regulation of *HRK* expression

**Figure 7:**
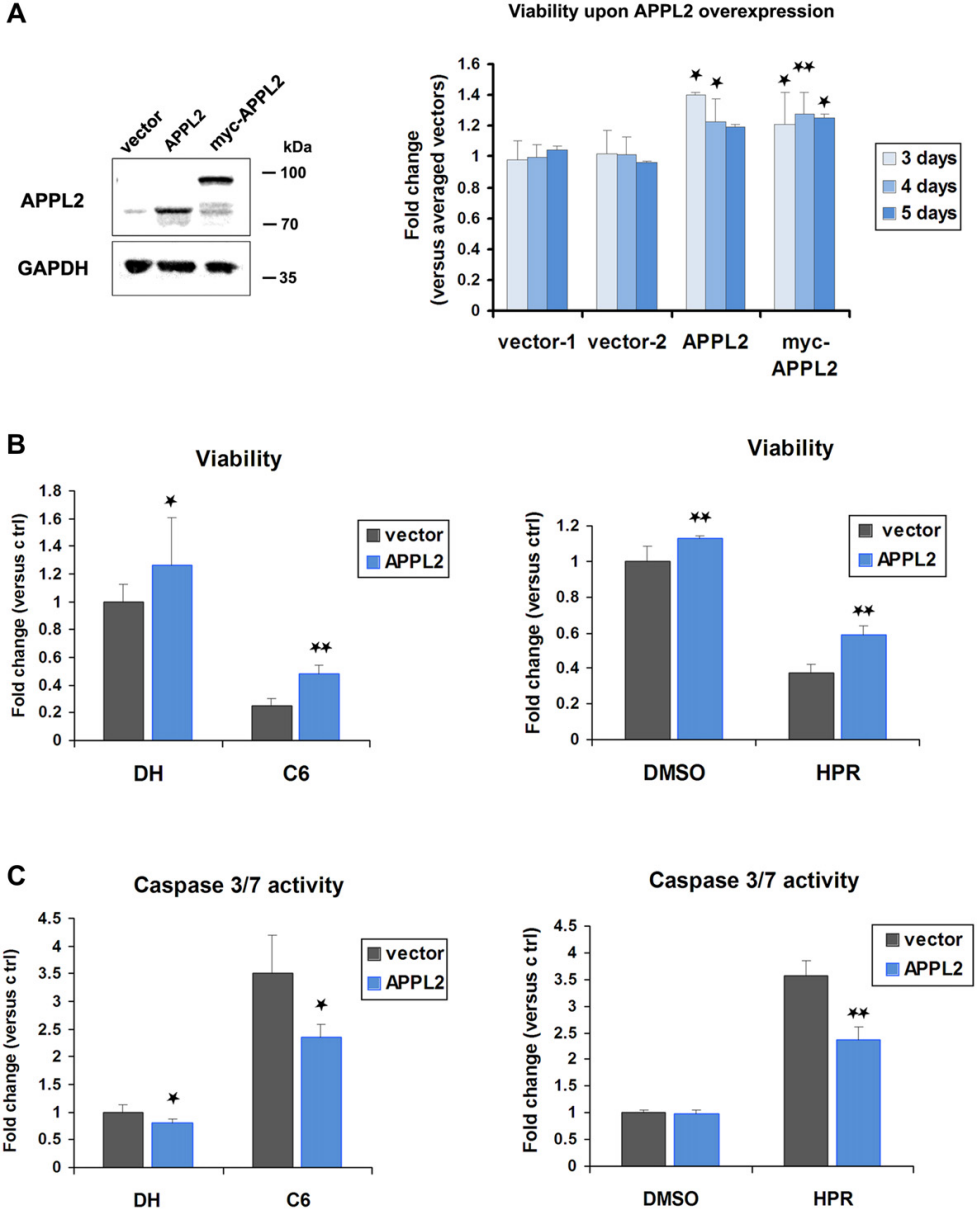
Overexpression of APPL2 protein enhances glioma cell survival and attenuates apoptosis. (A). Overexpression of untagged and myc‐tagged APPL2 in T98G cells under conditions of low serum availability (left panel; GAPDH used as loading control in Western blot). Cell viability (right panel) was analyzed 3, 4 and 5 days after transfection. The averaged values corresponding to 2 empty vectors (pcDNA3 and pcDNA3‐myc) at a given time point were set as 1. (B–C) The cell viability (B) and the activity of caspase 3 and/or 7 (C) were estimated in parallel upon transfection of T98G cells with empty vector or plasmid carrying myc‐tagged APPL2. One day upon transfection cells were treated either with 5 μM ceramide C6 (C6) or control dihydroceramide C6 (DH) (left panels), or with 2 μM HPR or DMSO (right panels) and analyzed 2 days later. The values for empty vector were set as 1. (A–C). The *p*‐values are marked with the asterisks (*p < 0.05, **p < 0.01).

To further confirm the functional relationship between APPL2 and HRK, we overexpressed APPL2 in T98G GBM cell line which has low endogenous amounts of APPL2 protein (Fig. 2A). In these cells, APPL2 overexpression potentiated cell viability under low serum availability (Fig. 7A), as also observed in LN229 cells (Suppl. Fig. 4). We further tested whether APPL2 overexpression increases viability of T98G cells treated with two apoptosis inducers: retinoic acid *p*‐hydroxyanilide (HPR) and ceramide C6. HPR‐induced apoptosis was previously characterized in GBM cell lines (Lytle et al., 2005), while ceramide was shown to induce the expression of *HRK* gene (Rizvi et al., 2011). We observed that high amounts of APPL2 protein potentiated resistance of cells to HPR‐ or ceramide‐induced apoptosis, as measured by increased cell viability (Fig. 7B) and reduced activity of effector caspases (Fig. 7C).

**Figure 8:**
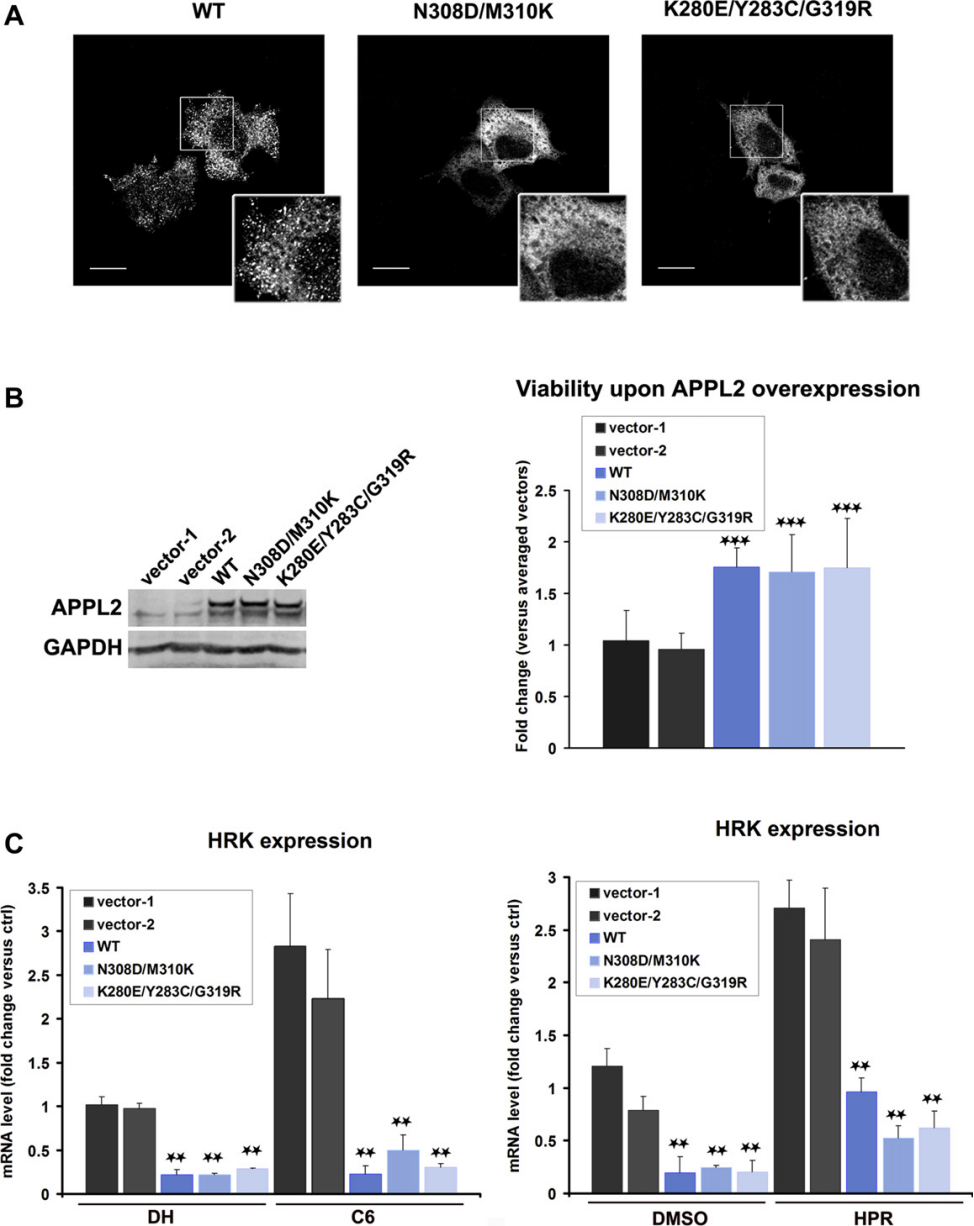
Endosomal localization of APPL2 is not required for its prosurvival activity and regulation of HRK expression. (A). HeLa cells were transfected with the myc‐tagged wild type (WT) or mutants of APPL2 (N308D/M310K, K280E/Y283C/G319R) and stained with an anti‐myc antibody. Scale bar, 20 μm (B). Overexpression of myc‐tagged wild type (WT) or mutants of APPL2 in T98G cells under conditions of low serum availability (left panel; GAPDH used as loading control in Western blot). Cell viability (right panel) was analyzed 3 days after transfection. The averaged values corresponding to 2 empty vectors (pcDNA3 and pcDNA3‐myc) were set as 1. (C). Expression of HRK was estimated by qRT‐PCR in T98G cells transfected with the indicated constructs and treated either with 10 μM ceramide C6 (C6) or dihydroceramide C6 (DH) (left panel), or with 2 μM HPR or DMSO (right panel) for 30 h. The *p*‐values were calculated upon comparison of each overexpressed construct to the corresponding empty vectors and were marked with the asterisks (**p < 0.01, ***p < 0.001).

To gain mechanistic insights into the observed cytoprotective effects, we tested whether the endosomal localization of APPL2 is important for its prosurvival activity. To this end, we constructed two mutants of APPL2, N308D/M310K and K280E/Y283C/G319R, based on the known mutations in APPL1 deficient in binding to Rab5 and thus not recruited to endosomal membranes (Miaczynska et al., 2004; Zhu et al., 2007). We verified that both mutants of APPL2 are indeed predominantly soluble in the cytoplasm and not localized on endosomes, in contrast to the wild‐type APPL2 (Fig. 8A). Interestingly, both mutants upon overexpression were equally potent in enhancing cell survival as the wild‐type protein (Fig. 8B). We further verified whether these cytoprotective effects of APPL2 overexpression correlated with decreased expression of *HRK* gene. Consistently, overexpression of the wild‐type or the mutants of APPL2 reduced *HRK* expression under conditions of HPR‐ or ceramide‐induced apoptosis (Fig. 8C). Cumulatively, these data argue that the endosomal localization of APPL2 is not required for its prosurvival activity and regulation of *HRK* expression in glioma cells.

## 4 Discussion

### 4.1 Influence of APPL2 expression on cell viability, apoptosis resistance and transformation abilities of glioma cells

Our results revealed that over 40% among 25 cases of human GBM appear to have a high level of APPL2 protein but not APPL1 when compared to non‐tumor tissue. Importantly, also in GBM samples containing light tumor infiltrate (the border region between non‐tumor tissue and tumor center) the level of APPL2 protein was significantly upregulated, although the number of investigated samples (7 cases) was small. A high level of APPL2 may contribute to the expansion of infiltrating tumor cells and favor some neoplastic properties of low‐grade brain tumors, since increase of its mRNA level was well noticeable in tumors of grade II and III (Sun et al., 2006). Such properties include elevated proliferation rates and resistance to apoptosis, ability to migrate and invade surrounding tissue, but not so much microvascular proliferation and angiogenesis, which are mainly the features of grade IV tumors (Brat and Mapstone, 2003).

In a model of two glioma cell lines, we demonstrated that silencing of APPL2 expression decreased their viability, apoptosis resistance and transformation abilities. In LN229 cell line, lower viability was evident only under limited serum availability, while in case of U87MG cells, it was also visible in regular serum concentration, however to a smaller extent. Importantly, growth factors are likely to be limited *in vivo* inside the nervous system parenchyma, where the infiltrating tumor cells have to survive and proliferate at high rate, thus *in vitro* studies under low serum availability are of physiological relevance. It is possible that in cultured LN229 cells the abundance of serum growth factors can completely overcome the need for the functional APPL2 protein to support cell viability. Moreover, the two cell lines may have different requirements for survival and APPL2 silencing could affect the signaling from growth factors that are more important for viability of U87MG than LN229 cells. In fact, the presence of different growth factor receptors on the plasma membrane of these cell lines was estimated by the antibody arrays (Stommel et al., 2007). U87MG appears to have detectable levels of EGFR, PDGFRβ, VEGFR2 and MET, while LN229 cells carry AXL, EGFR, EPHA2, ERBB2 and ERBB3 on their surface.

The effect of knockdown of APPL2 protein on decreased cell viability was at least partially due to enhanced caspase activity and stimulated apoptosis. Our results support a hypothesis that the high level of APPL2 protein in tumor cells may favor their resistance to apoptosis under limited growth factor availability. In accordance with our data, the study with antisense morpholinos revealed that knockdown of APPL1 or APPL2 triggered a massive apoptosis and resulted in embryonic lethality of zebrafish (Schenck et al., 2008). Similarly, knockdown of APPL1 in the Xenopus endoderm caused strong apoptosis in endodermal organs (Wen et al., 2010). Surprisingly, the embryonic development of mice was unaffected by the lack of *APPL1* gene (Tan et al., 2010). Unfortunately the *APPL2* knockout mice are not yet available to evaluate the role of APPL2 in mammalian development. Instead, silencing of APPL2 expression had no obvious effect on the viability of mouse embryonic fibroblasts under normal culture conditions (Tan et al., 2010), similarly to our results in glioma LN229 cells.

Long‐term silencing of APPL2 expression inhibited growth in soft agar even under regular serum concentration, in both investigated cell lines. The colony formation in soft agar requires growth under stringent anchorage‐independent conditions and is considered to be the characteristic of transformed cells. It is possible that the negative effect of silencing of APPL2 expression on growth in soft agar, as well as on tumor growth *in vivo*, was exclusively dependent on the inhibition of cell proliferation or enhancement of apoptosis. On the other hand, the effect on growth in soft agar was observed in both cell lines under regular serum concentration, raising the possibility that also other molecular mechanisms regulating anchorage‐independent growth (beside proliferation, survival and apoptosis) can be affected by knockdown of APPL2 protein.

### 4.2 Mechanisms responsible for enhanced cell death upon silencing of APPL2 expression

Our results demonstrating enhanced expression of proapoptotic genes *UNC5B* and *HRK* upon knockdown of APPL2 protein (Fig. 5) support the hypothesis that APPL2 may play a beneficial role in GBM development, through apoptosis prevention. The defects in apoptosis signaling and the augmented survival pathways are common features of GBM (Krakstad and Chekenya, 2010).

UNC5B is a member of the dependence receptor family, comprising transmembrane receptors that transduce prosurvival signals when engaged by a ligand, but emit proapoptotic signals in its absence. The disruption of dependence receptor signaling has been implicated in carcinogenesis (Goldschneider and Mehlen, 2010). Netrin‐1, the ligand for UNC5B, is an axon guidance molecule that plays also a crucial role during neuroblastoma development as well as in colorectal and breast tumorigenesis, by regulating apoptosis (Delloye‐Bourgeois et al., 2009; Fitamant et al., 2008; Mazelin et al., 2004). Different types of tumors and cancer cell lines display autocrine production of netrin‐1, thus blocking UNC5B‐induced apoptosis (Delloye‐Bourgeois et al., 2009; Fitamant et al., 2008). On the other hand, in some cell lines the overexpression of UNC5B is able to significantly enhance the apoptosis (Wang et al., 2009). One possible interpretation of our findings envisages that the high level of UNC5B expression upon knockdown of APPL2 protein could surpass the level of netrin expression, resulting in the presence of ligand‐free UNC5B receptors transducing proapoptotic signals. Such possibility would be supported by our findings that the levels of netrin‐1 mRNA remained unchanged in LN229 cells upon APPL2 depletion (data not shown) when the expression of *UNC5B* gene was increased Fig. 5. In addition, the presence of recombinant netrin‐1 in the medium significantly blocked the proapoptotic effect initiated by APPL2 silencing (Fig. 6B). We cannot however exclude that the other UNC5 family members may also contribute to apoptosis regulation in glioma cells. Therefore, further systematic measurements of protein levels of netrin ligands and UNC5 receptors upon modulation of APPL2 protein amounts will be required to uncover the exact relationship between APPL2 and UNC5 receptors.

HRK is an apoptosis‐facilitating protein, able to bind and inactivate the anti‐apoptotic partners Bcl2 and Bcl‐x (Inohara et al., 1997). Induction of HRK expression in neurons, primary hematopoietic cells and immortalized cell lines takes place upon deprivation of their requisite growth factors (Harris and Johnson, 2001; Imaizumi et al., 1999; Sanz et al., 2000, 2001; Wakabayashi et al., 2002). Sensory neurons from *HRK* knockout mice are less sensitive to apoptosis induced by NGF withdrawal (Coultas et al., 2007), while transient overexpression of Hrk induces cell death of sympathetic neurons (Harris and Johnson, 2001; Imaizumi et al., 1999; Putcha et al., 2001). In our experimental model, the enhanced expression of HRK correlated with the decreased cell viability and accelerated cell death. Moreover, overexpression of APPL2 reduced mRNA level of HRK, concomitant with increased resistance of cells to apoptosis, arguing that HRK abundance is an important factor contributing to prosurvival effects of APPL2.

In summary, our data demonstrate for the first time an upregulation of APPL2 protein in human tumor samples. We show that modulation of APPL2 level in glioma cells changes the expression of genes responsible for cell death induction, *HRK* and *UNC5B*, suggesting one possible molecular mechanism by which APPL2 may enhance tumor cell growth and apoptosis resistance. Intriguingly, these activities appear independent of endosomal localization of APPL2 and therefore could be mediated by its nuclear or cytoplasmic, but not endosomal, binding partners. Among others, APPL proteins interact with the nuclear proteins, such as nucleosome remodeling and histone deacetylase complex NuRD (Banach‐Orlowska et al., 2009; Miaczynska et al., 2004) or the multifunctional transcriptional regulator Reptin (Rashid et al., 2009). In the cytoplasm, APPL1 binds TRAF2 adapter protein which affects NF‐κB‐dependent transcription (Hupalowska et al., 2012). It is possible that some of these known binding partners may contribute to APPL2‐mediated regulation of *UNC5B* and *HRK* expression. Even though the precise molecular details of such regulation remain to be further studied, our data support an emerging notion that some multifunctional endocytic proteins may contribute to the regulation of gene transcription as well as tumorigenesis.

## Author contributions

BP designed and performed most experiments and analyzed the data; MBO, MTT, KM, MM^2^ and MM^1^ participated in designing or performing some experiments; GD helped with the pathological analysis of glioblastoma IHC; BP and MM^1^ wrote the manuscript. All authors approved the final version of the manuscript.

## Supporting information

The following is the supplementary data related to this article:

Supplementary data

## Supplementary data 1

### 1.1

Supplementary data related to this article can be found at http://dx.doi.org/10.1016/j.molonc.2012.08.003.
