# Supplementary material for: Multifunctional protein APPL2 contributes to survival of human glioma cells
Source: Mol Oncol. 2012 Sep 5;7(1):67–84. doi: 10.1016/j.molonc.2012.08.003 (PMC3553582; doi:10.1016/j.molonc.2012.08.003)
Supplement: Supplementary file 1 — Supplementary data [file MOL2-7-067-s001.pdf]

## Supplementary data

**Suppl. Table 1. The sequence of oligonucleotides used for silencing of APPL2 expression with the Block-it Lentiviral RNAi Expression System.**
The table summarizes the sequences (sense strand) of the custom-designed oligonucleotides targeting APPL2: APPL2-sh #1, APPL2-sh #2 and APPL2-sh #3 as well as the corresponding scrambled (scr.) control sequences.

**Suppl. Table 2. The primary antibodies used in Western blotting analysis.**
The table summarizes the primary antibodies used in this study for Western blotting analysis, including the catalog number and the vendor.

**Suppl. Table 3. The sequence of primers used for RT-PCR.**
The table summarizes the sequences of the primers designed with Primer Express 3.0 software and used further in the RT-PCR reaction with the Kapa Sybr Fast ABI Prism qPCR Kit. The last column includes the predicted size (in base pairs) of the PCR products.

**Suppl. Table 4. Clinical information concerning human glioblastoma (GBM) cases.**
The table summarizes the brain location and the recurrent history, if relevant, of the GBM cases used in this study. The age (in years) and sex of patients are also included (F, female; M, male). Each case number corresponds to the number in Fig. 1.

**Suppl. Table 5.**
The statistical significance of the viability data shown in Fig. 2 B, C. The significant *p*-values are written in italics.

**Suppl. Fig. 1. APPL2 mRNA is overexpressed in brain tumors of various histogenesis and grade.**
Oncomine microarray data analysis for APPL2 mRNA expression in brain tumors vs. non-tumor tissue is shown on the graphs. Two studies are included, Sun et al., 2006 (graphs on the left) and Bredel et al., 2005 (graphs on the right) comprising glioblastoma (GBM), oligodendroglioma (OD), anaplastic astrocytoma (AA) and oligoastrocytoma (OA) samples. The *p*-values were estimated by the Oncomine software, the log2 medians are represented by the horizontal lines.

**Suppl. Fig. 2. Silencing of APPL2 expression decreases viability of U87MG cells under regular serum availability.**
The bars represent the viability of U87MG cells transfected with APPL2 siRNAs and grown under regular 5% serum for 5 or 6 days (two sources of siRNA were used, as in Fig. 2). The error bars represent the standard deviation and the *p*-values were marked with the asterisks (* $p<0.05$, ** $p<0.01$).

**Suppl. Fig. 3. Silencing of APPL2 does not affect the levels of signaling molecules.**
Quantification of phospho-AKT, phospho-GSK3, phospho-ERK as well as p53 levels in the samples shown in Fig. 4A, using infrared imaging. The error bars represent standard deviation from 2 or 3 experiments.

**Suppl. Fig. 4. Overexpression of APPL2 protein enhances survival of LN229 cells.**
The untagged or myc-tagged APPL2 was overexpressed in LN229 cells under conditions of low serum availability. **(A).** The phase-contrast photographs of cells 4 days upon overexpression. **(B).** The Western blot analysis of APPL2 and GAPDH levels. Low exposure of APPL2 blot shows the difference between the endogenous and overexpressed levels. **(C).** The results of the cell viability assay that were calculated as in Fig. 3. The averaged values corresponding to 2 empty vectors (pcDNA3 and pcDNA3-myc) at a given time point were set as 1 (control). The error bars represent the standard deviation and the *p*-values were marked with the asterisks (** $p<0.01$).

# Suppl. Table 1

| **Oligonucleotide name** | **Sequence** |
|---|---|
| **APPL2-sh #1** | GGATCTCACAGAAGTAAGCAC |
| **APPL2-sh #2** | GCTGGTTACCTTAATCTTAGA |
| **APPL2-sh #3** | GAACCAGCATTCAGAAGTTTAA |
| **APPL2-scr. #1** | GACAGCAGAACCCGAATGTAT |
| **APPL2-scr. #2** | GGTCATTATCTCGGATTACAT |
| **APPL2-scr #3** | GAAATTGCACGGACCTATTAAA |

# Suppl. Table 2

| **Target** | **Cat. no** | **Vendor** |
|---|---|---|
| **β-actin** | A5441 | Sigma-Aldrich |
| **AKT** | 2920S | Cell Signaling |
| **phospho-AKT (S473)** | 9271S | Cell Signaling |
| **clathrin heavy chain (CHC)** | 610499 | BD Transduction Lab. |
| **EEA1** | 610457 | BD Transduction Lab. |
| **EGFR** | 610016 | BD Transduction Lab. |
| **phospho-EGFR (Y1173)** | 558382 | BD Pharmingen |
| **ERK1/2** | 9107S | Cell Signaling |
| **phospho-ERK (T202/Y204)** | 9101S | Cell Signaling |
| **GAPDH** | sc-25778 | Santa Cruz Biotech. |
| **GSKα/β** | 44-610 | Invitrogen |
| **phospho- GSKα/β (S21/9)** | 9331S | Cell Signaling |
| **lamin A/C** | sc-7292 | Santa Cruz Biotech. |
| **myc tag** | 05-419 (clone 9E10) | Millipore |
| **p53** | sc-126 (clone DO-1) | Santa Cruz Biotech. |
| **PTEN** | 9188S (clone D4.3) | Cell Signaling |
| **Rab5** | 18211 | Abcam |
| **α-tubulin** | T5168 | Sigma-Aldrich |

# Suppl. Table 3

| **Gene** | **Forward primer** | **Reverse primer** | **Product size** |
|---|---|---|---|
| **UNC5A** | CATCACCAAGGACACAAGGTTTGC | GGCTGGAAATTATCTTCTGCCGAA | 125 bp |
| **UNC5B** | GGGCTGGAGGATTACTGGTG | TGCAGGAGAACCTCATGGTC | 155 bp |
| **UNC5C** | GCAAATTGCTGGCTAAATATCAGGAA | GCTCCACTGTGTTCAGGCTAAATCTT | 114 bp |
| **DCC** | AGCCAATGGGAAAATTACTGCTTAC | AGGTTGAGATCCATGATTTGATGAG | 126 bp |
| **NTN1** | TGCAAGAAGGACTATGCCGTC | GCTCGTGCCCTGCTTATACAC | 108 bp |
| **NTN3** | GACTGTGACTCGCACTGCAAA | ACCTGCACCGCATAGTCCTT | 86 bp |
| **NTN4** | ACTCCAGTCCTTTTCCATGCA | TGTATCGTATGGTGGTGACAAAGC | 68 bp |
| **B2M** | TGGAGGCTATCCAGCGTACT | TGAAACCCAGACACATAGCA | 100 bp |

| | | | |
|---|---|---|---|
| | C | ATTC | |

## Suppl. Table 4

| Case no. | Location | Recurrent | Age / Sex |
|---|---|---|---|
| **GBM – light tumor infiltrate** | | | |
| 1 | temporal | | 68/ F |
| 2 | frontal | Yes, from oligodendroglioma | 33/ F |
| 3 | frontal | Yes | 43/ F |
| 4 | occipital | | 73/ M |
| 5 | frontal | Yes, from astrocytoma | 47/ M |
| 6 | temporal | | 55/ M |
| 7 | frontal | Yes, from astrocytoma | 36/ F |
| **GBM – tumor center** | | | |
| 8 | unknown | | 62/ M |
| 9 | temporal | | 59/ F |
| 10 | temporal | | 68/ M |
| 11 | unknown | | 83/ M |
| 12 | unknown | | 56/ M |
| 13 | temporal | | 55/ F |
| 14 | occipital | | 81/ F |
| 15 | temporal | | 73/ M |
| 16 | parietal | | 56/ M |
| 17 | parietal | | 65/ M |
| 18 | unknown | Yes, from astrocytoma | 49/ F |
| 19 | unknown | | 55/ M |
| 20 | unknown | | 51/ F |
| 21 | frontal | | 59/ M |
| 22 | unknown | | 65/ M |
| 23 | occipital | | 67/ M |
| 24 | frontal | Yes, from oligodendroglioma | 33/ F |
| 25 | temporal | | 68/ F |

## Suppl. Table 5

| **LN229 cells** | | |
|---|---|---|
| | **2 days** | **3 days** |
| **APPL2si #1** | *p= 0.0022* | *p= 0.0079* |
| **APPL2si #2** | *p= 0.0022* | *p= 0.0043* |
| **APPL2si #3** | p= 0.1320 | *p= 0.0043* |
| **APPL2si #4** | p= 0.0649 | *p= 0.0043* |
| **U87MG cells** | | |
| | **3 days** | **5 days** |
| **APPL2si #1** | *p= 0.0317* | *p= 0.0022* |
| **APPL2si #2** | *p= 0.0317* | *p= 0.0022* |
| **APPL2si #3** | *p= 0.0317* | *p= 0.0022* |
| **APPL2si #4** | *p= 0.0079* | *p= 0.0022* |

# Suppl. Fig. 1

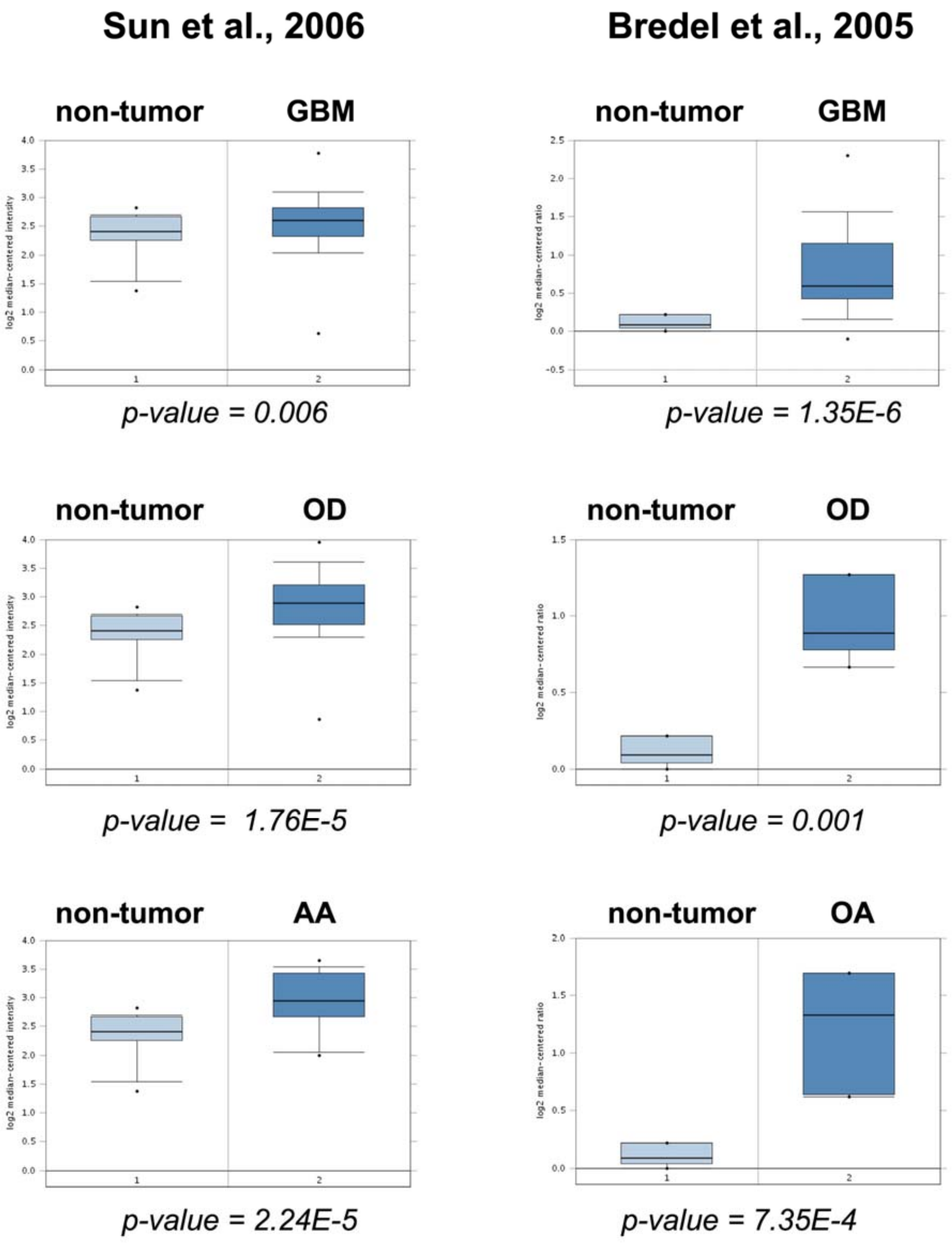
Sun et al., 2006
Bredel et al., 2005
non-tumor
GBM
log2 median-centered intensity
p-value = 0.006
non-tumor
GBM
log2 median-centered ratio
p-value = 1.35E-6
non-tumor
OD
log2 median-centered intensity
p-value = 1.76E-5
non-tumor
OD
log2 median-centered ratio
p-value = 0.001
non-tumor
AA
log2 median-centered intensity
p-value = 2.24E-5
non-tumor
OA
log2 median-centered ratio
p-value = 7.35E-4

# Suppl. Fig. 2

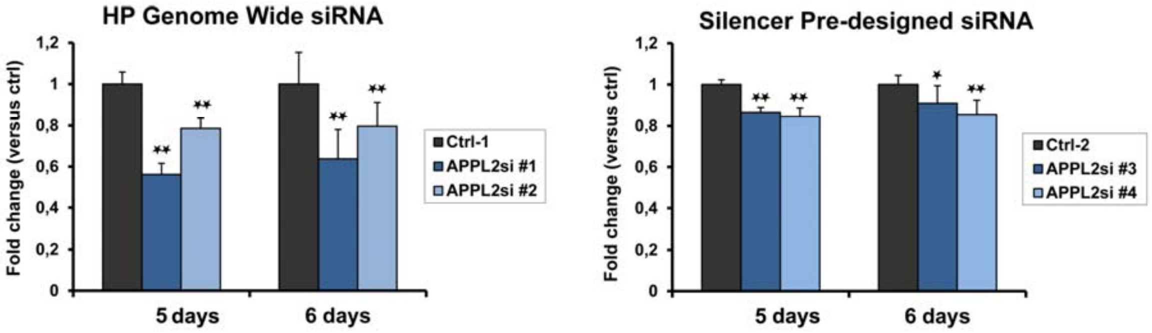
HP Genome Wide siRNA
Fold change (versus ctrl)
1,2
1
0,8
0,6
0,4
0,2
0
5 days
6 days
Ctrl-1
APPL2si #1
APPL2si #2
Silencer Pre-designed siRNA
Fold change (versus ctrl)
5 days
6 days
Ctrl-2
APPL2si #3
APPL2si #4

# Suppl. Fig. 3

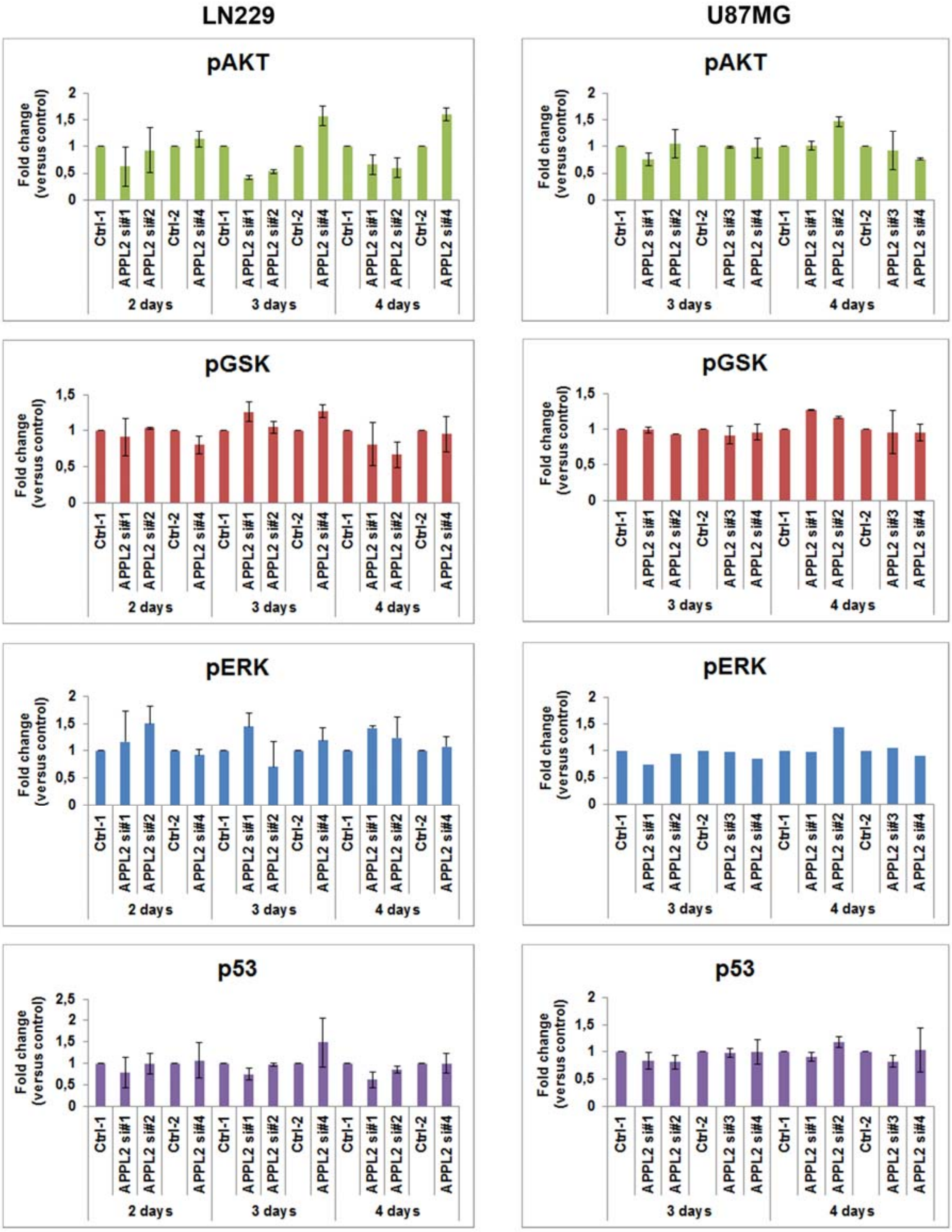
LN229
U87MG
pAKT
pGSK
pERK
p53
Fold change (versus control)
Ctrl-1
APPL2 si#1
APPL2 si#2
Ctrl-2
APPL2 si#3
APPL2 si#4
2 days
3 days
4 days
0
0,5
1
1,5
2
2,5

# Suppl. Fig. 4

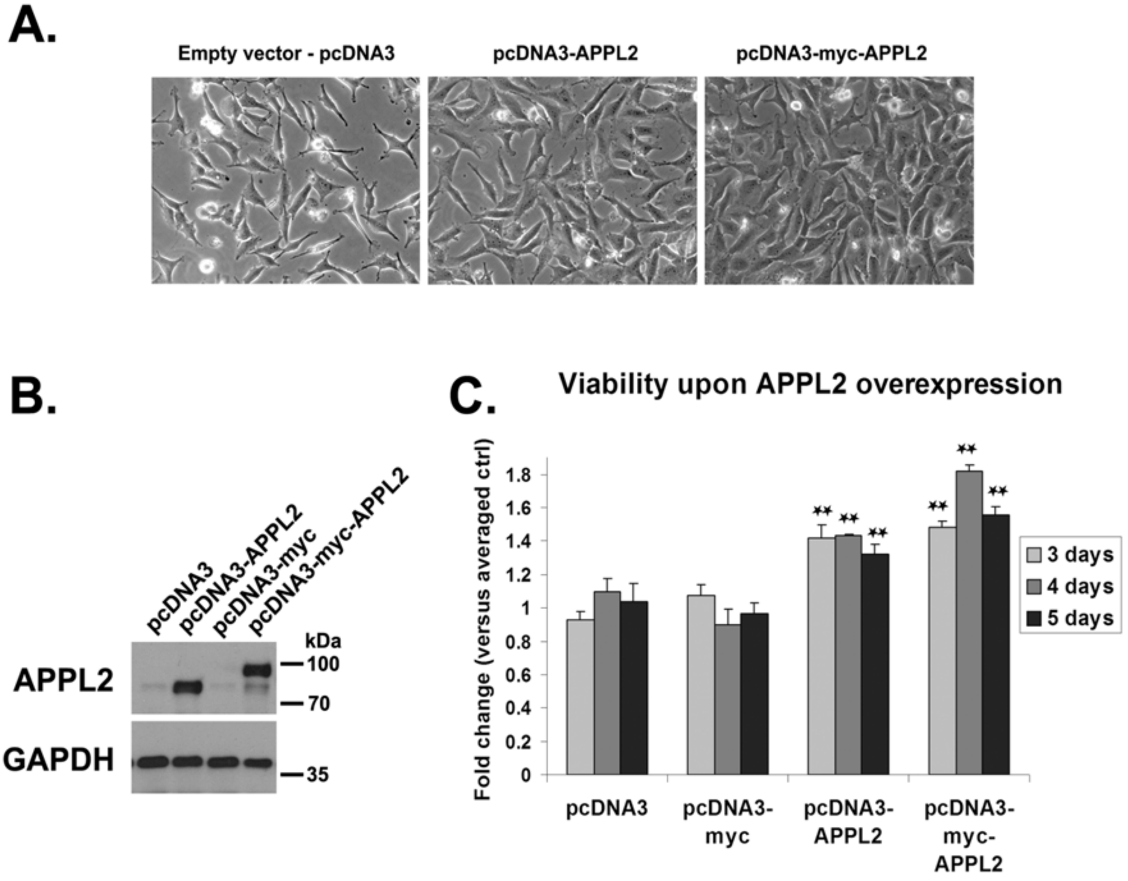
A.
Empty vector - pcDNA3
pcDNA3-APPL2
pcDNA3-myc-APPL2
B.
pcDNA3
pcDNA3-APPL2
pcDNA3-myc
pcDNA3-myc-APPL2
kDa
100
70
35
APPL2
GAPDH
C.
Viability upon APPL2 overexpression
Fold change (versus averaged ctrl)
1.8
1.6
1.4
1.2
1
0.8
0.6
0.4
0.2
0
**
3 days
4 days
5 days
pcDNA3
pcDNA3-myc
pcDNA3-APPL2
pcDNA3-myc-APPL2
